# The Bottleneck Metaphor of Leadership Culture: How Shared Understandings About Leadership Develop in Groups and Impede Diversity and Effectiveness of Leaders

**DOI:** 10.3389/fpsyg.2021.635751

**Published:** 2021-02-25

**Authors:** Muaz Özcan

**Affiliations:** Department of Psychology, Koç University, Istanbul, Turkey

**Keywords:** leadership emergence, organizational culture and climate, cultural change, leadership metaphors, bottleneck effects, diversity in leadership, leadership crisis

## Abstract

There are two big problems related to leadership today: unequal representation and high failure rates among leaders. This conceptual paper argues that commonly shared values, assumptions, and beliefs about leadership, i.e., universal leadership culture, are the common cause of both problems. After the concepts and levels related to leadership culture were explained, we introduce a multilevel, multi-actor process model named the bottleneck metaphor of leadership culture. This metaphor describes how leadership cultures are co-constructed by multiple actors based on their involvement in leader selection and reproduce themselves in groups over time based on emergent leaders' characteristics. Next, a diagnostic tool called “the leadership mirror” is proposed for organizations that want to assess their leadership culture's current state as a starting point for further interventions. Specific suggestions are made for various actors, ranging from individuals to organizations, for their possible roles in preventing undesired leadership cultures.

## Introduction

“One of the key problems today is that politics is such a disgrace, good people don't go into government. #WhyImGettingIntoGovernment” Donald Trump, 45th President of the USA.

“People want an authority to tell them how to value things, but they choose this authority not based on facts or results. They choose it because it feels authoritative and familiar.” Michael Burry, the first investor to recognize and profit from the American Mortgage Crisis in 2008.

Leadership is a significant factor in the success of groups of people in many levels, from small teams to companies, countries, and international organizations (Hogan et al., 2018). Today, there are two perpetuating problems of leadership. The first one is that toxic, abusive, and destructive leaders keep getting selected and followed despite the harm they cause (Lipman-Blumen, 2005, 2006; Padilla et al., 2007). Besides rarely leading their groups to their objectives in a sustainable manner, such leaders cause distress and negative consequences for many of their followers (Kiliç and Günsel, 2019). Although there are some successful leaders, leadership failures abound; research shows that leadership failure rates are much higher than ideal (Aasland et al., 2010; Hogan et al., 2010). The second issue is the inequality in representation in leadership positions (Bebbington and Özbilgin, 2013). Despite societal shifts in recent years, leadership is still exclusive and mostly reserved for a few elites like rich and privileged white men (Eagly and Chin, 2010; Randsley de Moura et al., 2018).

The common point of these two issues is persistence. Their existence continues despite all the efforts and resources spent to prevent them, such as leadership development programs in organizations and equality measures taken by the governments (Bullough and de Luque, 2015; Hogan et al., 2018). Why do these issues persist despite all efforts to eradicate them? What do individuals and organizations do to eliminate them and alleviate the inequality and ineffectiveness associated with leadership?

This conceptual paper provides answers to these questions. It uses literature from various fields and disciplines, such as industrial and organizational psychology, business and management, political science, sociology, and economics. The paper's main argument is a gestalt of shared values, assumptions, and beliefs about leaders and leadership endorsed by many humans. In return, this universal leadership culture causes and maintains these two seemingly unrelated problems.

People do not make leadership-related decisions in social isolation (Lord et al., 2020). Therefore, to fully understand these decisions and their roles in leadership-related problems, we need to start thinking about the leadership cultures surrounding such judgments (Day et al., 2014). Furthermore, the current issues of unequal representation and high leadership failures oblige us to identify the mechanisms that produce leadership cultures (Hogan et al., 2018). This objective requires a broad perspective to consider the complex, multi-actor nature of the co-construction of leadership cultures over time (Castillo and Trinh, 2018) and on different levels ranging from team to universal (Lord et al., 2020) based on the characteristics and decisions of the multiple actors involved in recruitment processes (April et al., 2010).

Studies investigating the existing leadership-related assumptions and beliefs are still mostly limited to the individual level and focused on how these affect individuals' leadership-related decisions (Lord et al., 2020). In addition to that, most of these studies ask how people judge others', but not their own, leadership prospects based on the perceived fit to leadership-related values, assumptions, and beliefs (Epitropaki et al., 2017).

However, individuals' assessments of self-compatibleness to leadership roles are equally important as their judgments about others regarding leader emergence (Hoyt and Murphy, 2016; Epitropaki, 2018; Aycan and Shelia, 2019). Hence, this paper aimed to discuss the characteristics of underlying leadership cultures that affect self- and other-directed leadership judgments that lead to leader emergence in groups. Furthermore, research on how assumptions and beliefs take shape and form on the individual or shared level is still nascent (Day et al., 2014; Wellman, 2017; Acton et al., 2019). The current paper aimed to provide theoretical answers to how leadership cultures take shape and change over time. It also discusses how organizational leadership cultures can be improved based on different actors' decisions and behaviors that lead to leadership emergence (April et al., 2010).

The current paper first defined leadership culture and explained it on several levels: individual to universal (or global). Then, the possible ways that leadership cultures might influence leadership-related decisions and outcomes are reviewed. Next, a multi-actor, multilevel process model, the “bottleneck metaphor of leadership culture,” is proposed. It describes how leadership cultures are co-constructed and reproduced over time, perpetuating the two previously mentioned problems. Afterward, a diagnostic approach, “the leadership mirror,” is described for organizations that want to assess their leadership culture's current state as a starting point for possible interventions. The section before the closing section includes several suggestions for various actors (i.e., existing leaders, followers/possible future leaders, and organizations) about developing better leadership cultures. The paper concludes with a discussion of potential implications of the new metaphor for future research directions.

## The Concept of Leadership Culture

Leadership culture is defined as the shared values, assumptions, and beliefs about leaders and leadership that help members of an organization decide and behave accordingly in leadership-related matters (Day et al., 2014). In other words, it is a shared understanding of ideal and typical leaders. These include the spoken and unspoken norms that determine how leaders should behave and who are expected and allowed to be the group's leaders.

The concept has its theoretical roots in the theory of leader categorization (Lord et al., 1984). This well-known theory asserts that individuals hold assumptions and beliefs, namely, implicit leadership theories (ILTs), about leaders and leadership (Day et al., 2014; Lord et al., 2020). Leadership culture forms when many organization members share similar assumptions and beliefs about leaders and leadership (Day et al., 2014).

Although its original conceptualization refers to the shared understandings of leadership on the organizational level (Day et al., 2014), the concept applies to groups at any level in theory. For example, within the scope of a well-recognized research project called *GLOBE Study of Leadership* (House et al., 2004), researchers modified the concept of ILTs to address leadership values share*d at the national level. T*he resulting construct was called “culturally endorsed implicit leadership theories” (CLTs) and was used to measure and compare different countries' leadership cultures.

However, CLTs based on the individual-level construct of ILTs might not fully capture the whole of leadership culture. In addition to shared values about leadership included in the GLOBE Study, leadership culture also encompasses assumptions and beliefs about who should be the leaders and how leaders are expected to behave (Day et al., 2014). However, these are not included in CLTs (Hanges and Dickson, 2004; Javidan et al., 2010). Still, the GLOBE Study is critical because its findings indicate that distinct leadership cultures may exist on the national level while uncovering six dimensions of leadership-related values that are common cross-culturally, which implies some degree of universality in the human understanding of leadership (Hanges and Dickson, 2004; Day et al., 2014).

Universally shared understandings about leadership may include some of the deepest, almost instinctual presuppositions about leadership. These must be the hardest to challenge and change. Possible examples may comprise commonly observed associations of leadership with dominance (Maner, 2017), power difference (Vanderslice, 1988; Wolff and Keith, 2019), hierarchy (O'Toole et al., 2002), gender (Schein et al., 1996), charisma (Menges et al., 2015; McKee et al., 2017), and perks and privileges like fat paychecks and high levels of social prestige (Regan, 2016). Although there are variations, such pairings with leadership are observed in almost every culture (Den Hartog et al., 1999). In terms of leadership understanding, these consistencies among different nations and societies show that humans might be sharing a universal (or global) leadership culture.

On the more macro levels, universal and national leadership cultures can encourage specific categories of individuals (Epitropaki, 2018) to aspire for leadership positions more than others (Davies et al., 2017; Badura et al., 2020). They can also intimidate certain groups of candidates. Women and introverts, people with disabilities, and those with disadvantaged socioeconomic backgrounds that do not match the collective expectations for leaders and leadership might be more worried about leading (Bebbington and Özbilgin, 2013; Epitropaki, 2018; Aycan and Shelia, 2019). Besides influencing self-assessments, shared understandings about leadership can also affect opinions regarding the suitability of different categories of candidates in leadership roles and cause stereotypes to occur (Lord et al., 2020). Due to the anticipation of these stereotypes, various groups of people, such as males and females, might feel that they fit and perform differently under different leadership cultures than each other (Eagly et al., 1992, 1995).

Similarly, in more micro scales, judgments about the same targets' (self and others) appropriateness for the different leadership positions and roles might change due to the different leadership cultures perceived to be surrounding the seat in question (Aycan and Shelia, 2019). Candidates who are unwilling to accept offers of particular leadership positions might respond differently to an offer of another rank in the organization or similar positions in other companies (Epitropaki et al., 2017; Zaccaro et al., 2018). Therefore, what matters for each candidate might be the degree of the perceived fit between their values, assumptions, and beliefs and the perceived leadership culture of the particular leadership position (Day et al., 2014). Candidates might reject offers based on the long working hours and the anticipated possibility of work–life imbalance (Aycan and Shelia, 2019) because of the expectations mandated by the surrounding leadership cultures to leaders. Like any other beliefs and assumptions, these beliefs should also be considered as included in the perceived leadership culture of the position in question (Day et al., 2014).

The same logic for micro levels also applies to differing suitability judgments for different categories of people other than the self in terms of the perceived fit between them and various leadership positions based on leadership cultures (Ayman and Korabik, 2010; Badura et al., 2020; Lord et al., 2020). For example, people tend to associate certain leadership positions more with male and female genders (Ayman and Korabik, 2010). A wide variety of personal and position-related factors can affect these fitness assessments, like the demographic and personality characteristics of targets and the role expectations and working conditions/hours for the leadership position (Ayman and Korabik, 2010; Badura et al., 2020; Lord et al., 2020). Consequently, people from different categories might be judged as more or less suitable for leadership roles surrounded by different leadership cultures (Ayman and Korabik, 2010).

Leadership culture is different from the traditional approaches to study cultural change, such as Schein's classic book on the role of leaders in the production of organizational culture (Schein, 2004) or Schneider's attraction–selection–attrition (ASA) model (Schneider, 1987) that explains the role of members' personalities in the organizational culture change. It is relatively freer from some of the criticisms directed toward Schein's and Schneider's works that criticize organizational culture as a too complex and comprehensive construct to be only explained by leaders' influences or the personalities of members (Hofstede, 1986; Edwards et al., 2006).

Instead of dealing with a broad concept of organizational culture with more comprehensive but more challenging to define boundaries, the idea of leadership culture is exclusively about how leadership is defined and understood and the what are the expectations for leaders in a group (Day et al., 2014). This way, it deals with a much less complicated and more easily definable construct than a concept like the organizational culture that includes much larger numbers of factors than leadership culture (Day et al., 2014).

Although it is more straightforward than the broader concept of culture, measuring leadership culture is still not an easy task (Day et al., 2014). When leadership culture is measured directly with self-report questionaries, organizational members may deliberately or unintentionally provide inaccurate answers. This problem occurs possibly due to the distinctions between espoused and in-use assumptions and beliefs (Argyris, 1976a, cited in Argyris, 1976b; Schein, 2004, cited in Schein, 2004) that shape the group's leadership culture. Espoused leadership theories represent the group's ideas about leadership, a state where group members collectively wish to be at (or pretend to wish) in leadership (Schein, 2004).

In contrast, in-use theories are the ones that are currently operating and affecting the leadership outcomes of the group (Schein, 2004). Ideally, these two are aligned, but measuring leadership culture may be especially difficult when they are not. Like people, organizations also want to appear socially desirable (Brown et al., 2006). Thus, value-laden declarations by organizations, such as value charts and vision and mission statements, might not always reflect the truths about their real leadership culture. Sometimes, organizational members might not even be aware that what they think they have does not match the leadership culture they have (Brown et al., 2006; Day et al., 2014).

## Leadership Climate

Leadership culture is defined and used together with a parallel construct: the leadership climate, which refers to how a given group of people perceive and experience their group's leadership culture (Day et al., 2014). It is an interface in which members use their assumptions and beliefs about leadership to perceive and interact with the collective's leadership culture that developed over time. In other words, the leadership climate is produced and experienced within each cross-section of time. In contrast, leadership culture is the longitudinally accumulated product of many interactions in past leadership climates (Day et al., 2014).

There is a recursive relationship between leadership culture and climate. Leadership culture, coupled with members' leadership-related assumptions and beliefs that they bring into the picture, serves as the underlying framework for the existing leadership climate perceptions (Day et al., 2014). In return, depending on the outcomes they produce, the current leadership climate contributes to the future developments and changes in the group's leadership culture through the related decisions and members' actions over time (Day et al., 2014).

As a construct based on perception, leadership climate is much more dependent on the current leaders' characteristics and the followers who interact with them (Day et al., 2014; Swart-Opperman and April, 2018). Thus, a leader who does not comply with the group's leadership culture can cause a group to experience an entirely different leadership climate than expected from its leadership culture. Members can also experience a misaligned leadership climate due to outside factors such as a societal-level crisis or insider myths produced *via* gossiping and inaccurate storytelling about leaders (Popper, 2012; Popper and Castelnovo, 2018; Wantaate, 2019).

On the other hand, leadership culture is produced by past leaders and followers who served in the organization over time; thus, it is much more rooted and resistant to change. It has a significant role in how the existing group members experience the leadership climate. However, the future leadership culture change can mostly come from the leadership-related decisions and actions taken in the current leadership climate (Day et al., 2014). Thus, the leadership climate is like the engine producing the change in the group's leadership culture in the long run. Any attempt to change the leadership culture should start by modifying the leadership climate in the desired direction. In other words, misalignments between leadership climates and cultures could be intentionally introduced to the systems to create a change in leadership cultures (Day et al., 2014).

The author of the current paper argues that any human group can have a distinct leadership culture and climate regardless of its size and function. Although leadership cultures at higher levels influence the lower levels, indigenous leader cultures and climates of the smaller subunits under the larger shared structures (i.e., organizational or national leadership cultures and climates) can still exist. For example, different business sectors operating in the same economy can have different leadership cultures. Furthermore, organizations from the same business sector located in different countries can share similar understandings about leadership due to the shared nature of the work they do.

Similarly, different departments and branches of organizations can have distinct leadership cultures and climates diverging from the organizational-level leadership culture due to the particular leaders in those sections. Lastly, the two concepts apply to the team level since different teams under the same corporate umbrella can have different leadership types and develop various leadership understandings (Scott et al., 2018).

**Figure 1:**
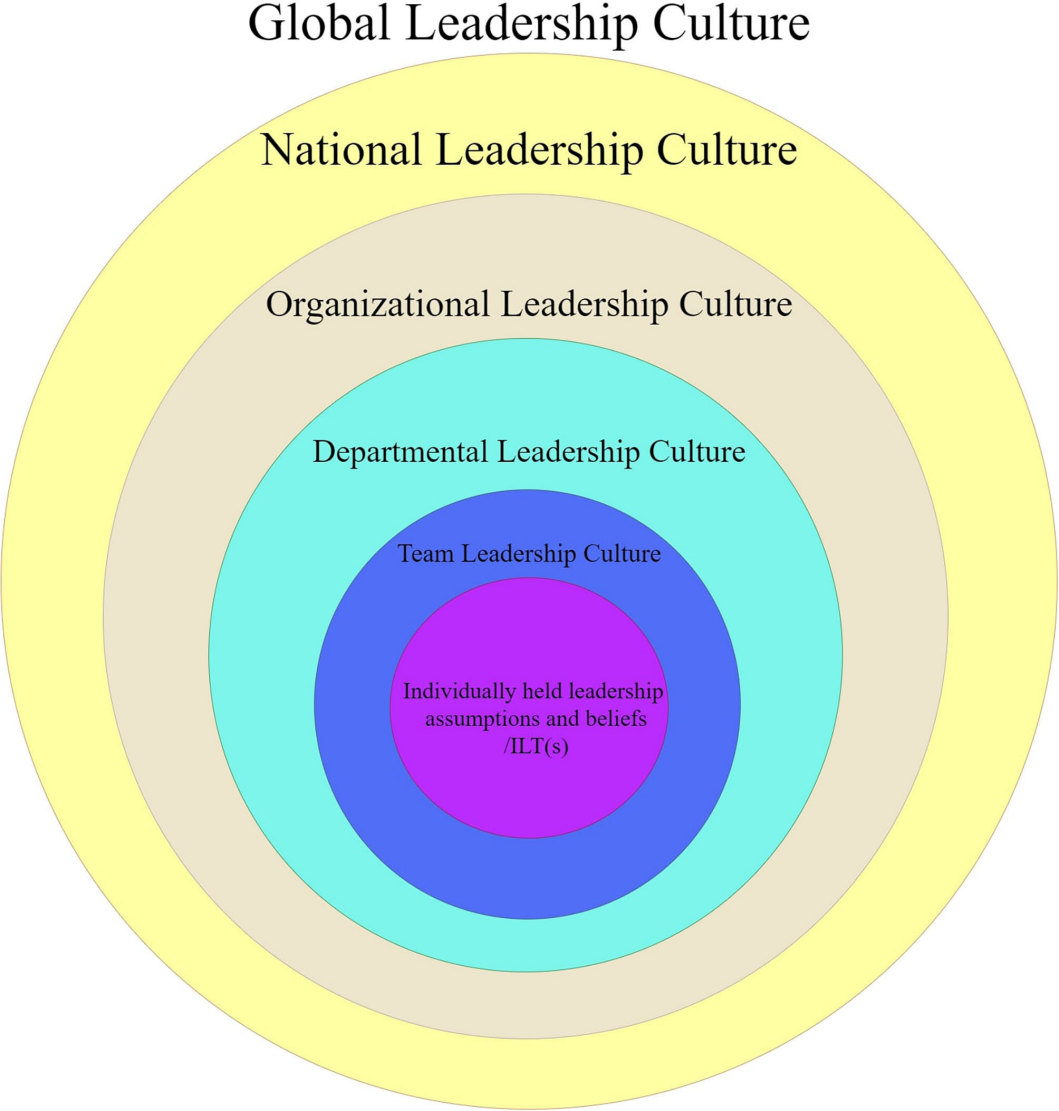
The different levels of leadership cultures and climates.

Figure 1 presents the different levels of leadership cultures and climates proposed in the current paper. Theoretically, leadership cultures and climates that exist at higher levels influence the leadership cultures and climates of the lower levels that they incorporate. Likewise, changes in the leadership cultures and climates at each level can trigger changes in their superordinate levels.

As the bottom line, the leadership culture and climate determine who will be the leaders or followers and what kind of leadership types a group endorses and gets (Tom, 1971; Schneider, 1987; Schein, 2004; Day et al., 2014). Thus, these two concepts might have theoretical links with critical group outcomes, such as leadership effectiveness, representation equality, and overall group performance. If human groups were rational organisms, group learning mechanisms due to negative or positive outcomes in terms of the group's benefits (Rescorla and Solomon, 1967) would improve any group's leadership culture over time. Unfortunately, the social and cognitive realities of humankind are not that simplistic. People themselves, who are the basic units of these group formations, are not entirely rational beings in their decisions and behaviors (Tversky and Kahneman, 1974).

## Possible Symptoms of Existing Leadership Cultures

There is no research that we know of which compiles and lists all the widely shared assumptions and beliefs about leadership. However, by looking at symptoms and figuring out what types of people are more likely to become leaders in the current conditions, it is possible to have a rough picture.

Indeed, some groups of people are represented more often in leadership roles than others (Eagly and Chin, 2010; Bebbington and Özbilgin, 2013). Individuals who are male, heterosexual, tall, attractive, and with privileged socioeconomic and racial backgrounds become leaders more often than other people (Ilies et al., 2004; Eagly and Chin, 2010; Bebbington and Özbilgin, 2013). Specific personality characteristics such as dominance and assertiveness increase individuals' chances to become leaders (Judge et al., 2009; Ensari et al., 2011). Other characteristics such as agreeableness and introversion decrease the odds (Judge et al., 2002; Dilchert, 2007; Spark et al., 2018).

Interestingly, having the so-called dark personality traits (narcissism, psychopathy, and manipulativeness) also increases the likelihood of becoming a leader (Babiak et al., 2010; Wisse and Sleebos, 2016). Narcissistic and psychopathic personality traits are about four times more prevalent among leaders than the general population (Babiak et al., 2010; Smith and Lilienfeld, 2013; Wisse and Sleebos, 2016; Landay et al., 2019). These findings indicate that some people are welcomed for leadership roles more than others.

Besides the unequal representation, other problems can also be traced back to today's universal leadership culture. It might also be the reason behind the high prevalence of unethical and exploitative leader behaviors and the alarming rates of failure among leaders (Aasland et al., 2010; Hogan et al., 2010). These performance-related issues might be due to the gap between characteristics that predict an effective leadership and the features that help to emerge as leaders (Hogan et al., 2018). Universal leadership culture seems to favor the latter more.

Research on leaders' effectiveness suggests that good leaders should be competent and good at communication and team building (Luthans, 1988; Dal Bó et al., 2017; Chamorro-Premuzic, 2019). They should be open to feedback and criticisms (Collins, 2001; Cain, 2012; Tost et al., 2013). Having integrity is a must for effective leadership (Hooijberg et al., 2010). Being too dominant or assertive is negatively related to leader effectiveness (Tost et al., 2013). Instead, researchers suggest a healthy mix of humility and motivation for leaders (Collins, 2001; Owens et al., 2015). They must listen to what others think and need and be willing to delegate when needed (Wang et al., 2014).

On the other hand, the literature on leader emergence depicts a very different picture (Hogan et al., 2010, 2018). Political ability and manipulativeness work better for success in emerging as a leader (Luthans, 1988). Compared to the effectiveness requirements, what is essential here is not necessarily being but appearing confident, calm, and motivated (Hogan and Kaiser, 2005). Some call this mixture of perceived characteristics required for political and business leaders to emerge as the fearless presence (Babiak et al., 2010) or the fearless dominance (Lilienfeld et al., 2012).

Having facial features perceived as dominance signaling and being tall and masculine are pros for emergence (Judge and Cable, 2004; Anderson and Kilduff, 2009; Antonakis and Dalgas, 2009; Ensari et al., 2011). Instead of the healthy blend of humility and motivation, people who are good at emerging have a mixture of charisma and motivation that helps them to be bold at claiming and getting into leadership positions (Collins, 2001). More puzzlingly, the candidates who are perceived to be extroverted, assertive, and dominant and the arrogant, selfish, and egoistic individuals appear as more leader-like in the eyes of other people (Boudreau et al., 2001; Spark, 2020).

To sum up, today's leaders, in many aspects, constitute a homogenous group to some extent. Many leaders share similar characteristics like narcissism, masculinity, and assertiveness (Epitropaki, 2018). They are also usually from similar and mostly specific socioeconomic status (SES) levels, ethnicities, genders, and sexual orientations (Bebbington and Özbilgin, 2013). Furthermore, a considerable portion of today's leaders fails in sustainably delivering positive results for the organizations they lead (Aasland et al., 2010; Hogan et al., 2010). Polls indicate that political and organizational leaders are among the least trusted professions by the public in almost every society for the last two decades, and the trust rates continue to decrease (Ipsos, 2019; Reinhart, 2020). At the same time, political discourses that emphasize populism and tribalism are dangerously on the rise and threaten the democracy in many countries of the world (Moffitt, 2016; Roth, 2017; Fukuyama, 2018).

All these problems about leadership that accentuate rather than be on the mend indicate that there might be a deep-rooted common cause that produces and maintains the undesirable outcomes. What is worse is that things are going even worse as time passes. Universal leadership culture, in other words the assumptions and beliefs about leaders and leadership shared by humanity, can be the underlying reason. These associations of being and looking leader-like might be deeply ingrained in the universal leadership culture. Even 5-year-old children's decisions reflected some of these when asked to select between the pictures of actual candidates running in the elections (Antonakis and Dalgas, 2009). They tend to choose dominant-looking candidates to be the captains of their imaginary boats.

It is not the case that only leadership scientists know the requirements of effective leadership. Laypeople also seem to be aware of these characteristics; when the qualities of ideal leaders are asked for, the answers resemble the findings of the research on effective leadership (Kouzes and Posner, 2010). However, candidates who make ineffective and unethical leaders are repeatedly chosen (Hogan and Kaiser, 2005; Lipman-Blumen, 2005, 2006; Padilla et al., 2007; Aasland et al., 2010; Hogan et al., 2010, 2018). This paradoxical situation again signals an underlying, probably unconscious, and deeply internalized factor that prevents us from making better decisions in leadership-related matters (Brunell et al., 2008).

The universal leadership culture might be the factor that causes people to select familiar and authoritative over factual and beneficial without realizing their roles in creating adverse leadership outcomes (Bligh and Kohles, 2012; Peus et al., 2012). Today, researchers know that power can corrupt (Giurge et al., in press), even at the neurological level (Hogeveen et al., 2014). However, it might be corrupting because of the deeply rooted assumptions and beliefs about leadership. If humans want better leaders than the current ones, the existing leadership cultures on almost all levels that are more welcoming to the characteristics related to success in emergence than effectiveness should be modified or replaced with better alternatives.

Exceptions of this need for leadership culture change only include a couple of particular leadership cultures. Some leadership cultures might need to be preserved due to their specific contexts that render authoritative and disciplined leadership necessary (Popper, 2012). For example, teams operating in the military, oil platforms, and other similar field settings that require quick decisions and executions might be better off with the existing leadership cultures.

## Bottleneck Metaphor of Leadership Culture

Readers might ask what are the origins of the leadership cultures that we have today and need to change so badly? Why did they develop into the way they are today? The current paper focuses more on how leadership cultures can be improved to provide more equality and effectiveness in leadership cadres. Before moving to the intended and directed changes, we would like to offer some possible answers to these questions.

There are three explanations that we can think of as to why today's universal leadership culture is more aligned with qualities for leadership emergence than leadership effectiveness. The first comes from the evolutionary perspective on leadership. According to this view, today's leadership emergence-related qualities were functional for survival in human groups' past environments (van Vugt et al., 2008). Thus, these were naturally selected and have evolved to become the qualities that people unconsciously search for when they judge potential leaders (White, 2011).

The second explanation, which complements the first, comes from the psychological perspective on leadership-related choices. According to the idea, followers took comfort in leaders by granting them the authority to deal with the world's uncertainties and unknowns on their behalf (Hogg and Adelman, 2013). People need leaders who appear confident, sure, and authoritative in uncertain times (Post, 1986). They hope that these strong, sometimes tyrannical leaders are more cunning and less merciful in dealing with outsiders and hostile groups than their relations with in-group members (Haller and Hogg, 2014). Hence, whenever the uncertainties increase, such as during crises and war times, public support rates for these leaders increase (Randsley de Moura et al., 2018).

The evolutionary explanation is problematic due to three reasons. Some people might defend existing leadership cultures and claim that we should preserve those. Because they are natural selection products, they must be optimal (Salter, 1995; White, 2011). Thus, the evolutionary explanation can quickly lead to the formation of naturalistic fallacies (Frankena, 1939), which many right-wing ideologues cling to Salter (1995), claiming that what is natural in terms of authority and leadership is equal to what is right (White, 2011). Naturally, this is not the fault of the evolutionary hypothesis *per se*, but its threat is real.

Secondly, not all products of evolutionary processes are functional; there can be mismatches between today's requirements and what was useful in the past (van Vugt et al., 2008). For example, in past environments, having a body that can hold extra reserves in the form of body fat would be advantageous in terms of survival (Power, 2012). However, in today's changed conditions, it might lead to obesity. Similarly, the authoritative, dominant, and masculine leaders could be what human groups needed in the past. However, they may have become obsolete in modern conditions (van Vugt et al., 2008). Alternatively, some evidence shows that instead of evolution, these might be the products of modernity and produced by cultural evolution after the advent of agriculture and large-scale societies (Garfield et al., 2020). In one way or another, today's business and work environments are too fast-paced, uncertain, and complicated for groups to trust and depend on a few persons' abilities and goodwill (Rodriguez and Rodriguez, 2015; Bawany, 2016).

Thirdly, theoretical arguments about the existence of group selection as a natural selection mechanism are not concluded yet (Jeler, 2015). Evolutionary mechanisms are supposed to work through individuals' survival and reproductive successes, not groups (Wade, 1978). As previously discussed, leadership culture is a group dynamic; thus, validation attempts of the existing leadership cultures based on the evolutionary explanations should be approached cautiously. If group selection exists, then it becomes evident that human social evolution is the most appropriate phenomenon for this mechanism to operate (Bowles, 2006). However, the claim of group selection leading to strong authoritarian leaders requires making weak assumptions and historical arguments about past environments of evolutionary adaptiveness (van Vugt et al., 2008).

The psychological explanation must also be approached with caution. It explains people's preferences for certain types of leaders in war and conflict situations. However, it does not explain why authoritative leaders still exist even during more prosperous and peaceful times. Besides, why these leaders are still common in more micro levels like team and organization levels, where there is no large-scale violence or wars, remains unanswered. Therefore, this explanation might be useful in explaining why certain types of leaders are preferred more at certain times. However, it does not validate the current leadership culture's assumptions and nullify the apparent need to change these.

The third explanation comes from an ecological perspective. According to this perspective (Powers and Lehmann, 2014), the agricultural revolution that happened around 10–13,000 years ago introduced authoritative and dominant leaders to human societies for the first time. Before the private ownership concept, there was no mechanism to coerce individuals to obey authority except physical force. People would simply leave the area to hunt and gather resources in other areas instead of tolerating dominance efforts plaguing a place (Garfield et al., 2020). However, the agricultural revolution introduced the concept of controlled access to resources, thus rendering domination to be possible. After thousands of years of coercion, humans might have developed familiarity with and fondness for hierarchical, authoritative, dominant, and masculine leaders (O'Toole et al., 2002).

Whatever the correct answer as to why people prefer certain kinds of leaders, the author of the current paper leans toward the idea that humans take their leader selection decisions based on familiarity. Even the artificial intelligence (AI)-based recruitment tool developed by Amazon Company had to be shelved after it started to favor typical candidates for technical jobs (Amazon ditched AI recruitment tool, 2018). The algorithm was designed in such a way that it could learn characteristic features of professions on its own using data available on the internet.

Unexpectedly, learning about job occupants' typical features led the program to make recruitment choices based on demographics; thus, it became discriminatory. The next section will discuss how leadership cultures can be intentionally changed toward more desired directions to obtain leadership cultures conducive to equality and effectiveness. It starts with a discussion on alternative strategies in terms of efficiency and feasibility.

### Changing the Leadership Culture

How can something as complex and deeply rooted as universal leadership culture with a perpetuating and persistent nature be changed? As mentioned in previous sections, anyone who wants to improve the leadership culture should change the leadership climate to create a misalignment between these two structures. The critical question here is, which is the best level to introduce misalignments to trigger a feasible, efficient, and sustainable change in leadership cultures at many levels most easily? Simultaneously targeting leadership cultures at all levels would, of course, be ideal. However, this strategy would not be feasible due to the massive amounts of resources required. We believe that the best option to start a full-scale change in the leadership culture at different levels is to target the organization's leadership climate and leadership culture.

Targeting only the universal-level leadership culture would not be realistic due to the massive scale of the required interventions. On the other hand, targeting national-level leadership cultures would require intervening in countries' politics. Thus, this strategy might face too much resistance from the existing political leaders like dictators, who are unwilling to give up their vast amount of power (Buchanan and Badham, 2020). Such leaders do not care about getting positive results for the people; all they care about is preserving their grasp on authority for as long as possible (Larcom et al., 2014).

For this purpose, some leaders even tend to engage in negative selection. They deliberately promote incompetent individuals to leadership positions and eliminate competent candidates to prevent contenders to their rule (Egorov and Sonin, 2011). Not all political leaders are dictators, of course, but as readers themselves may have noticed; the current political panoramas are not very promising. Most governments of today's world have started to look like “kakistocracies” (Abadjian, 2010; Okafor et al., 2014; Adams and Crosby, 2017). It is a term used for government systems controlled and ruled by the worst and least deserving, the most incompetent, and the least ethical members of society (Amorado, 2012).

On the other hand, organizational leaders are more open to changes if they know that the changes will bring in profits in the short term and sustainability in the long term (Kanter, 2003). Conveniently, change and innovation are frequently and seriously discussed topics in the business world (Dunphy et al., 2003; Arifin, 2020). Most humans living in modern societies work in various organizations. Among other mechanisms, people are mostly socialized to their societies' culture by their family members at a young age (Schein, 2004). If the organizational leadership cultures start to change, employees might socialize their children and young relatives to the new understandings of leadership that they are exposed to at work. Thus, some of the changes in the next-generation's leadership cultures on the national or even the universal level can be realized based on modifications made at today's organizational-level leadership cultures.

### Leadership Culture as Bottleneck

How the leadership cultures of organizations can be changed is an important question. We propose that, unless measures are taken against it, an organization's existing leadership culture at a given time point can act as a bottleneck. Getting rid of this bottleneck might be challenging. This challenge is because of its self-feedbacking nature, which possibly perpetuates the same assumptions that limit who can desire leadership positions and become the organization's leaders.

As the first principle of the bottleneck argument, everyone, regardless of whether they are insiders or outsiders to the group, should be considered as a potential candidate for the group's leadership positions in theory. Thus, like other selection processes in organizations (Truxillo et al., 2017), the candidate pool's initial diversity levels for leadership positions in terms of various individual difference variables must be very high. In principle, everyone, both the in-group and out-group members, can be considered as potential leaders for the group regardless of their characteristics.

However, individuals self-select themselves and become (or not become) applicants for each leadership position (Epitropaki, 2018). These choices are made based on the fitness that candidates anticipate between themselves and the leadership culture of the position that they perceive (Day et al., 2014; Lord et al., 2020). They reach some conclusions about the underlying leadership cultures by observing the leadership climates regarding these positions (Day et al., 2014). As discussed in previous sections, the most apparent leadership culture indicators are the existing leaders occupying the same or similar ranks and positions.

Candidates observe the existing leaders and, whether consciously or not, calculate an approximation of the position's leadership culture. Calculations are likely to be made based on perceived traits of the past candidates who were welcomed to the organization's leadership positions (Cantor and Mischel, 1977). The ones who feel that their characteristics are compatible should be the people who apply for the role. In contrast, others might withdraw if they think they are incompatible with the surrounding leadership culture (Day et al., 2014). Thus, possible effects on the candidates' self-nominations to leadership positions constitute why the leadership culture might act as a bottleneck that reduces the diversity in leadership positions. This part is the first stage of the bottleneck of leadership culture. In this phase, the existing organizational leadership culture acts as a bottleneck on the candidate's self-selection to compete for leadership positions.

In addition to affecting candidates' decisions to become applicants, leadership culture can also influence the authorities' decisions about which applicants to select and promote the group's available leadership positions. Like candidates, leaders, or judges who choose them, also have opinions about the group's leadership culture based on their observations on the current leaders' typical characteristics in these positions. Thus, the group's leadership culture acts as a bottleneck, affecting the group's leadership emergence processes again. This part is called the second stage of the bottleneck of leadership culture. In return, applicants appointed as the group's new leaders might, directly and indirectly, affect the leadership culture perceived by the candidates and judges in future selection scenarios. These long-term effects constitute the third stage of the bottleneck of leadership culture. In this phase, it reproduces itself through the leaders it allows to emerge and gets even tighter and more exclusive compared to its initial permeability.

Reproduction happens because the selected applicants become the new authorities who steer the group as expected from group leaders. They can now give directions, provide values, and modify the group's culture thanks to the new authority given to them. It is also possible that they would be heavily involved in selecting the group's future leaders; thus, there is always the risk of homophilic reproduction (McPherson et al., 2001). The term refers to some leaders' problematic tendencies of exclusively attracting and selecting or promoting candidates who are very similar to themselves in various individual difference variables (Samdanis and Özbilgin, 2020).

Even if they do nothing directly, by just being themselves, the existing leaders may affect the perceptions of whom and how the typical leaders of the group should be. Thus, they influence the perceived leadership climate and are considered the embodiments of the group's leadership culture. Consequently, leadership culture becomes restrictive to diversity in leadership positions not once but twice, affecting candidates' decisions to apply and leaders' decisions regarding selection or promotion. These restricting effects occur each time a leader selection procedure takes place. Over time, they accumulate and change the group's leadership culture used in future leader emergence processes; thus, it gradually gets less inclusive.

We decided to use the bottleneck metaphor to refer to how leadership culture can restrict who become leaders in a collective. The radius of its passing changes each time based on the characteristics of the individuals who could emerge as leaders. Inspiration for the metaphor came from the bottleneck and the founder effects (Templeton, 1980; Lande, 1988) from the population genetics literature (Templeton, 1980; Lande, 1988). Together, these two concepts explain evolutionary outcomes of sudden selection pressures, like natural disasters, that critically reduce a population's size between two generations and cause genetic pools to lose diversity over time (e.g., Garoff et al., 2020).

In this line of explanations, the bottleneck effect refers to sudden and arbitrary selection pressures and processes that determine who survives (or selected) and who does not. Examples of bottleneck events include earthquakes, floods, wildfires, and similar disastrous events, eliminating some of the population. The founder effect refers to the reduced genetic diversity of the next generations due to the bottleneck events. Bottlenecks and founder effects decrease the diversity of the genetic pool of population when the selection pressures, by sheer coincidence, cause only certain types of members to survive, reproduce, and form the new genetic pool. The bottleneck metaphor warns organizations that the leaders they choose today can both influence the leadership climates of today and the leadership cultures they will have in the future.

In social sciences, there were few incidents where similar metaphors refer to selection scenarios that are deemed dysfunctional because of their inability to provide desired and targeted outcomes. For example, Fishkin (2014) used the bottleneck metaphor to argue that selection procedures like current university entrance exams must be illegitimate and arbitrary. He argued that instead of achieving the intended purposes, they act as bottlenecks reinforcing the existing inequalities like underprivileged groups' lack of access to societal opportunity structures.

Just like the biological mechanisms explained previously, leadership cultures might act as bottlenecks that create the selection pressures and determine who becomes the next generation of leaders and who does not? Leadership cultures and climates on higher levels, like universal and national, constitute the luggage people carry with them to their organizations. Every person who joins an organization brings their ideas, or mental models, about how things are and how they should be in the world around them (Schein, 2004). Mental models are acquired through the socialization processes that people go through and tend to reflect the reinforced assumptions in their families, national cultures, or previous groups (Schein, 2004). Through intensive socialization processes, mental models become deeply ingrained. Any changes in them require unlearning what people already internalized (Schein, 2004). Thus, even when there are efforts and precautions at the organizational level to increase leadership cadres' diversity and effectiveness, due to the strong influences of deeper mental models acquired through previous socializations (Hofstede, 1986; Schein, 2004), these attempts might still look like swimming against the current. Unless organizational-level leadership culture is continuously monitored and supported in the desired directions, positive effects are likely to vanish quickly (van den Brink, 2020).

The characteristics of the individuals who were able to emerge as leaders under the existing assumptions and beliefs about the organization's leadership pose the future selection pressures in the following leader selection and emergence scenarios. Thus, if they are not checked continuously and intervened in, leadership cultures tend to produce even more exclusivist versions of themselves that constitute further bottlenecks in the future. In other words, whether the current leadership culture constitutes arbitrary bottlenecks for certain groups of people must be checked. Otherwise, organizations cannot achieve maximum potential in terms of leadership diversity. This issue can pose profound fairness and effectiveness problems. Moreover, when the effects of the universal and national leadership cultures on the organizational leadership cultures through the luggage the members bring into organizations are considered, the issue becomes even more problematic. Higher-level leadership cultures might further contribute to bottlenecks that cause the persistence of unequal representation and high leadership failure rates in organizations.

**Figure 2:**
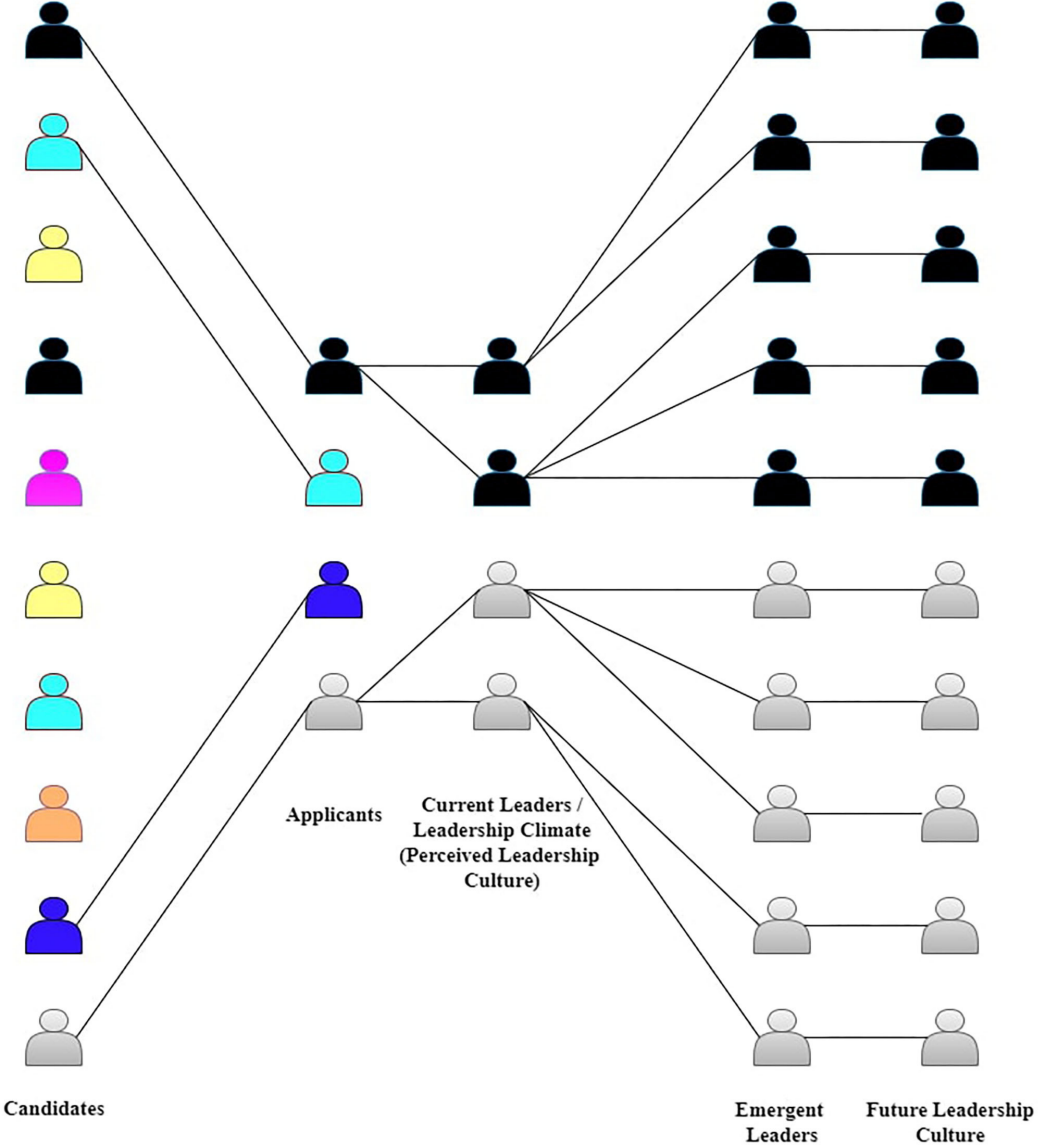
Illustration of how a given group's leadership culture acts as a recursive bottleneck decreasing the diversity that exists in the group's leadership positions over time.

On the macro levels, the gains made at the universal and national levels achieved in the past few decades in terms of leadership effectiveness, equality, and diversity should be kept under constant monitoring for their continuity due to the harmful effects of cultural bottlenecks. Similarly, organizations should consider the sustainability of the effects in the long run rather than merely implementing short-term intervention programs like diversity training with impermanent effects (van den Brink, 2020). Figure 2 depicts how a given group's leadership culture acts as a recursive bottleneck decreasing the diversity that exists in the group's leadership positions over time.

Besides restricting diversity, which is beneficial for a group's overall performance by creating competitive advantages (Peteraf, 1993), bottlenecks can also negatively affect a group's leader effectiveness. Individuals who feel entitled to lead or see themselves over group norms are more likely to snatch available leadership positions. They are more inclined to apply and they more easily get selected than the responsible candidates who think a lot before acting (Grijalva et al., 2015).

These snatchers include individuals with dark traits (i.e., certain types of subclinical narcissism, psychopathy, and manipulativeness), high levels of risk-taking tendencies, and personality characteristics like dominance and assertiveness (Ensari et al., 2011; Wisse and Sleebos, 2016). Being bold is a critical factor in order to emerge as a leader (Nevicka et al., 2011). Thus, some people are more likely to pass the bottleneck, although they do not deserve to lead more than the other candidates (Lanaj and Hollenbeck, 2015). These include men who feel more confident within the current leadership cultures than women (Chamorro-Premuzic, 2019), adding to the unequal representation issue. They also encompass some of the less competent candidates who are not aware of their shortcomings as much as more competent individuals are (Dunning, 2011), contributing to the ineffectiveness problem. As previously discussed, commonly shared assumptions and beliefs about leadership and leaders, namely, the universal- and national-level leadership cultures, are usually more encouraging and accepting of these people. Higher-level leadership cultures legitimize some people (e.g., males over females) and traits more than others (e.g., masculinity more than femininity; Ayman and Korabik, 2010). These discrepancies in legitimacy perceptions cause power and influence to be more easily given to typical rather than atypical leaders (Samdanis and Özbilgin, 2020). As legitimate power is highly required for introducing changes in organizational cultures (Clement, 1994), vicious cycles that produce unequal leadership and ineffective leaders are more likely than virtuous cycles even in organizations where these were initially in good shape.

## Leadership Mirror

Since the idea of changing a leadership culture intentionally in the desired direction is still in its early phases, we cannot provide an exact recipe for potential interventions. However, we can provide a diagnostic tool as a starting point for organizations that want to assess and change their leadership cultures. We named this method the “leadership mirror.” Its basic premise is to identify groups of candidates who want and who do not want to take leadership positions in an organization in general or for a particular leadership position. These groups should then be compared to each other based on the chosen individual characteristics to see whether the existing leadership culture acts as a bottleneck that discriminates in undesired ways. These comparisons can be based on the demographics, personality traits, individuals' assumptions and beliefs about an ideal leadership, or on various amalgams. The aim is to draw an approximation of an organization's leadership culture based on the group differences between the candidates who want or who do not want to apply.

To do that, organizations can ask the same question to these people in different ways: whether they want to lead in this organization in general or in specific leadership positions. The use of available agentic constructs in the literature related to leadership selection decisions, like worries about leadership (WAL) (Aycan and Shelia, 2019) and motivation to lead (MTL) (Chan and Drasgow, 2001), might be useful in obtaining more nuanced assessments of leadership cultures.

WAL measures three different types of commonly observed worries that individuals experience when they imagine themselves being in a leadership position in the future (Aycan and Shelia, 2019). These different concerns include worries about failure, harming oneself and others, and not being able to balance work, family, and other requirements of life spheres.

MTL measures three types of motivations that people have that make them aspire and pursue leadership positions (Chan and Drasgow, 2001). The first motivation is wanting to lead because of liking the concept of leadership and seeing it as a part of the self-identity (affective-identity MTL). The second dimension is wanting to lead because of a sense of duty and obligation felt toward the community (social-normative MTL). The third dimension is wanting to lead because of not being too calculative about the possible pros and cons related to leading.

The WAL questionnaire's referent structure is suitable for modifying the survey questions to refer to the exact leadership role that is asked of candidates to consider (Aycan and Shelia, 2019). MTL questions are, on the other hand, more about the participants' general trait-like motivational attitudes toward the idea of them becoming a leader (Chan and Drasgow, 2001; Bobbio and Rattazzi, 2006; Badura et al., 2020). Compared to the MTL, the WAL is more of a state than a trait. Thus, it might be more suitable for use as a tool within the scope of the proposed application of the leadership mirror perspective in measuring leadership culture. However, by adding one question asking whether the participant wants the leadership position in question or not, the MTL can also be a useful proxy measurement of leadership cultures.

The WAL and MTL are reported to be orthogonal, meaning they are independent of each other in terms of the variances they explain in leader selection decisions. Thus, using WAL's and MTL's constructs together as proxy measurements of leadership cultures would be the best practice of the leader mirror approach. Previous theories and findings indicate that whether they assess themselves or others, people have both avoidance and approach motives in their leadership-related judgments (Kark and Van Dijk, 2007; Van Quaquebeke et al., 2010). Moreover, such decisions have two aspects: they can simultaneously depend on value congruence and incongruence between individuals' values and those perceived to be dictated by the position (Schuh et al., 2018). Using the WAL and MTL together coincides with both types of such motives, thus capturing a more holistic picture of the leadership culture.

Furthermore, both constructs' multidimensional nature lends themselves to even more detailed assessments of leadership cultures. For example, people's scores on the subdimensions of WAL and MTL can be used in conjunction with each other to identify candidate profiles by using the latent profile analysis (LPA) method (Howard and Hoffman, 2018). This method would give a more detailed picture of the types of employees who want and who do not want a particular leadership position due to the leadership culture they perceive. Alternatively, the scores on the subdimensions of these two questionnaires can be used to compare the profiles of the existing leaders to those of the candidates and applicants (Auvinen et al., 2020).

Using the leadership mirror approach, organizations can find answers to what categories of people want or do not want to lead within them. Then they can learn the reasons behind these by applying qualitative research methods such as focus group interviews. They can use the information obtained to modify the leadership cultures or perceptions about them in desired ways. Hence, they can turn the leadership positions more alluring for the targeted candidates and drive others away, even from applying. This way, they can save time and money and increase the applicant pool's quality while monitoring future leadership culture bottlenecks.

## Implications and Future Directions

Different parties who have roles in forming and modifying organizational leadership cultures might want to know what they can do to prevent negative leadership cultures. In this section of the paper, we will provide suggestions for each of these actors.

Individuals might think they are powerless and helpless against the larger collective structures such as leadership culture (Kossek et al., 2017). Atypical candidates must not forget that their decisions and actions impact them and the collective opinions about the groups they might represent in leadership positions (Samdanis and Özbilgin, 2020). Compared to the more typical candidates, being selected as leaders will also be much more challenging for them (Phelan et al., 2008). Suppose they ever become leaders against all the odds. In that case, their mistakes will be evaluated and judged much more harshly than the tolerance shown to more typical leaders by communities (Rudman and Glick, 2001). In addition to these, there is always the risk of turning into a somewhat typical leader who perpetuates the existing obsolete leadership culture existing in human groups (Harvey, 2018; Baykal et al., 2020).

Still, there is hope. In other words, all possible candidates for leadership positions, almost everyone besides the current leaders, should be aware of the power and influence of their decisions or the absence of them about pursuing and claiming leadership positions on the surrounding leadership cultures. In a leadership position, the presence of an individual, or lack thereof, is vital to breaking the presumptions about who typically can be and who cannot be in this role (Eagly, 2018a). Even getting selected is not necessary to shatter groundless and harmful associations in people's minds regarding who can be and who cannot be the leaders. Self-nomination and application to leadership positions are also challenging these on their own (Mariani et al., 2015). Considering the increased diversity rates in the candidate pool of the following congressional elections, Clinton might have sparked a social movement among women, youth, and other underrepresented groups by losing to Trump in 2016 (Cargile et al., 2020).

Existing leaders must be aware of their crucial direct and indirect gatekeeper roles in creating and reproducing bottlenecks of leadership culture (Broockman, 2014). It should always be kept in mind that leaders' influences within the scope of bottlenecks are not limited to what they do or whom they select and promote. These also include the things that they choose not to do and the characteristics of the candidates that they did not consider for the vacant seats. Moreover, how followers and potential candidates perceive leaders' and organizations' behaviors and decisions is essential (Jacobsen and Bøgh Andersen, 2015). Thus, current leaders should ensure that they precisely convey the messages they want to communicate regarding their organizations' leadership culture to candidates and applicants.

Organizations should acknowledge three things about the leadership culture that can impose bottlenecks for much-desired leadership diversity and effectiveness. Suppose the group's leadership culture is permitted to develop and change in its natural course. In that case, bold candidates will fill vacant positions sooner or later, despite their possible shortcomings (Nevicka et al., 2011). The leadership cultures of the organizations will increasingly become inconducive to diversity and effectiveness over time. Thus, changes in them should be continuously monitored and managed. Human resource specialists working in organizations must ensure that they always have a highly diversified group of leaders. Expecting a homogenous group of leaders to select and empower a diverse group of next-generation leaders is paradoxical (Bebbington and Özbilgin, 2013). The same warning also applies to the degree of homogeneity in leader selection committees, even for the times that these collectives consist only human resource specialists and do not include leaders (Daskalova, 2018, 2019).

Secondly, organizations should not solely focus their attention and efforts on one part of the big picture of the leadership problems (Allio, 2007). Leadership culture is developed and changed co-constructively. Thus, organizations must prioritize developing better leadership systems and cultures simultaneously while aiming for better leadership (Padilla et al., 2007; Day et al., 2014). Instead of attending only to separate parts of leadership problems, like unequal representation and lack of effectiveness, they should work on improving both individual members and organizational structures (Schein, 2004; Day et al., 2014; Kossek et al., 2017).

Thirdly, companies must acknowledge that just because they think they have a healthy leadership culture, it does not mean that candidates perceive it similarly (Schein, 2004; Day et al., 2014; Jacobsen and Bøgh Andersen, 2015). Having a conducive leadership culture in terms of diversity and leader effectiveness is not enough; candidates must be perceiving it in the same sense (Schein, 2004; Day et al., 2014). This point is critical. The order of events described in the bottleneck metaphor indicates that sustained diversity in the candidate pools is the primary condition to avoid overly exclusive leadership cultures harming organizations. Organizations should guarantee that the right 678 messages about the leadership culture are broadcast and transmitted. One rogue leader, or some baseless myths and rumors about the group's leaders and leadership structures, can contort the candidates' perceptions about the group's leadership culture in many negative ways (Foster, 2000; Popper, 2012; Day et al., 2014; Popper and Castelnovo, 2018; Wantaate, 2019).

Governments and other policy-making agencies also have their responsibilities in creating, maintaining, and developing leadership cultures. Policies like quotas can be beneficial to increasing diversity, but to reach their maximum potentials, they should be supported with the right kind of leadership cultures (Pande and Ford, 2011; Bullough and de Luque, 2015; Mölders et al., 2018).

Otherwise, the places reserved for the categories of people with specific demographics, like parliament seats allocated to women, might be filled with individuals whose other characteristics, like personalities, are compatible with the existing obsolete leadership cultures (Fitzsimmons, 2012; Harvey, 2018; Baykal et al., 2020). Solutions to leadership diversity and effectiveness problems require radical changes in the prevailing leadership cultures (Al Ariss et al., 2014). Candidates have multiple combined characteristics like gender, sexual orientation, race, age, class, and ideology. Thus, instead of focusing on each variable in isolation, sensible intervention policies must be based on the intersectional understanding of individual differences (Kamasak et al., 2019).

Furthermore, people should stop trying to fit women and other less represented groups in leadership positions designed according to the mainstream understandings about leaders and leadership (Braun et al., 2017; Kossek et al., 2017). Existing leadership cultures have mostly produced toxic, abusive, and homogenous groups of leaders; thus, these need to change, not the other way around (Beard, 2017; Hogan et al., 2018; Chamorro-Premuzic, 2019). Besides, binary definitions of gender and sexual orientation are outdated now. Thus, using binary conceptualizations in the twenty-first century to generate solutions for gender-related inequalities is a bit self-handicapping and an inconsistent strategy (Eagly, 2018b; Bae et al., 2019).

Lastly, we want to discuss the bottleneck metaphor's implications for current and future research about leadership. The bottleneck metaphor indicates that there can be possible biases, especially in the findings of leadership effectiveness studies that sample existing leaders. Thus, such studies might be prone to various self-selection and survivorship biases (Brown et al., 1992; Epitropaki, 2018). Thus, researchers must cautiously approach these findings. The topic of reluctant leaders remains severely under-investigated in the leadership literature (Epitropaki, 2018; Aycan and Shelia, 2019). Future studies must extend the leadership emergence literature to encompass not just applicants but candidates as well.

Consequently, researchers who study leader effectiveness should acknowledge that their participants are limited to those who select themselves as leadership candidates. Thus, the results might not reflect the full picture of leader effectiveness. Current leaders might not be the best sources to investigate the negatives about companies' existing leadership cultures or the unfairness in the leader selection processes. After all, the same bottlenecks that they asked to identify and discuss are what produced them.

Naturally, discussing all the possible questions about leadership culture and its bottleneck functions in leader selection scenarios in just one conceptual paper is not an accomplishable task. Readers should treat this article as an igniter that hopes to draw attention to some of the less investigated leadership literature topics. These include the impacts of shared assumptions and beliefs about leadership, reluctant leaders, and possible biases in the findings of leadership studies. The paper will serve its purpose if it ignites further discussions and inspires new studies about leadership cultures at different levels and avenues where leadership exists.

Due to space-related concerns and lack of available findings, some issues related to the concepts and ideas presented in this paper were not covered. How leadership cultures at different collective levels form and influence leader selection decisions other than the organizational level need more discussion and research. Similarly, essential questions like how differently the bottlenecks operate in different leadership avenues, such as political leadership, business leadership, and leadership in non-governmental and voluntary organizations, remain unanswered.

The mechanisms of the bottleneck metaphor of leadership culture discussed for organizations might operate differently in political settings since relationships between followers and leaders differ from each other in each arena. Political leaders are more distant to their followers compared to organizational leaders who closely and bidirectionally interact with their followers almost every day; thus, the symbols and meanings are much more important in political compared to organizational leadership (Popper, 2012).

Future studies can also investigate the “as is” and “should be” forms of leadership cultures at different levels to identify the differences between the existing and the desired leadership culture. This approach is the same as what the Globe Study did to measure cultural value dimensions. Unfortunately, they did not apply the same method to the measurements of leadership-related values in this very comprehensive research project (House et al., 2004).

Lastly, the factors causing the differences between the perceived and the actual leadership culture and the effects of such gaps on group outcomes promise lucrative research areas. We already suggested some mechanisms: leaders who do not act in parallel with the leadership culture that the organization wants to adhere to or the myths and rumors created by gossiping and inaccurate storytelling about leaders (Popper, 2012; Popper and Castelnovo, 2018; Wantaate, 2019). Future studies on these and other mechanisms that possibly cause differences between the perceived and the actual leadership culture are severely needed.

Some of the ideas discussed in this paper can also pave the way for a more situational understanding of leadership emergence. Leadership researchers and practitioners, for a relatively long time, are aware that leadership effectiveness depends not only on the leaders but also on the conditions and settings that they lead in Fiedler (1966), Sims et al. (2009), and Thompson and Glaso (2018). These include individual characteristics of their team members and team-level features (Swart-Opperman and April, 2018). We should also start acknowledging that leadership emergence-related judgments of individuals, when they evaluate themselves and others, are too situational and contingent to settings. Thus, human resource professionals and researchers must not be quick to assume that candidates who do not want a specific leadership position lack desire and motivation for leadership. Their discomfort might be specific to the position; in other roles or organizations, they might be in peace with the idea of them being leaders. Hence, new theories of leader emergence that better incorporate the possible contingencies must be developed.

## Conclusion

To sum up, collective assumptions and beliefs about leaders and leadership, in other words leadership culture, might cause unequal representation in leadership positions and high leader failure rates. They might be resistant to change because they simultaneously affect both the existing and potential leaders' decisions in leader emergence processes.

Change in universal leadership culture can be accomplished most efficiently by changing the current leadership cultures existing at organizational levels. However, an organization's leadership culture acts as a bottleneck that only allows candidates who perceive themselves and are perceived by others as compatible. Hence, these tend to reproduce themselves and the leadership cultures seen at higher levels. It is possible to change a leadership culture by introducing intentional misalignments between the leadership culture and climate. The most obvious indicators of the leadership culture underlying the leadership climate in an organization are the perceived commonalities among the existing leaders.

Organizations need to know how different groups of candidates perceive their leadership cultures. They must figure out who is or who is not attracted to their leadership positions. They can use the questionnaires of the available agentic constructs related to leadership emergence to obtain more detailed answers to these questions. Lastly, leadership cultures have their implications for many different actors on various levels. Thus, the multilevel, multi-actor nature of co-construction of change in leadership cultures must not be ignored when theories and interventions are developed.

## Author Contributions

MÖ is the sole author of this paper. Thus, MÖ completed all the tasks, including the literature review, argument generation, manuscript writing, figure drawing, and reference listing and checking.

## Conflict of Interest

The author declares that the research was conducted in the absence of any commercial or financial relationships that could be construed as a potential conflict of interest.
